# Prevalence and prognostic significance of malnutrition in early-stage multiple system atrophy

**DOI:** 10.3389/fnut.2023.1248349

**Published:** 2023-11-22

**Authors:** Shirong Li, Lingyu Zhang, Yanbing Hou, Tianmi Yang, Chunyu Li, Qianqian Wei, Ruwei Ou, Xueping Chen, Huifang Shang

**Affiliations:** ^1^Laboratory of Neurodegenerative Disorders, Department of Neurology, Rare Diseases Center, National Clinical Research Center for Geriatrics, West China Hospital, Sichuan University, Chengdu, China; ^2^Department of Neurology, Guizhou Provincial People’s Hospital, Guiyang, Guizhou, China

**Keywords:** multiple system atrophy, controlling nutritional status score, malnutrition, survival, cohort study

## Abstract

**Background:**

Malnutrition is associated with poor survival in some diseases. However, the nutritional status in multiple system atrophy (MSA) is unknown, and the significance of malnutrition for the prediction of mortality in MSA has not been well established.

**Objective:**

We aimed to determine the prevalence of malnutrition and the prognostic value of malnutrition in patients with early-stage MSA.

**Methods:**

Patients diagnosed with early phase MSA (disease duration<3 years) were recruited, and they were followed every year until May 2023. The nutritional status of patients with MSA was assessed using the Controlling Nutritional Status (CONUT) score and Geriatric Nutritional Risk Index (GNRI). Kaplan–Meier survival analysis and Cox regression model were used to assess the prognostic value of malnutrition in MSA.

**Results:**

A total of 224 patients with probable MSA (106 MSA died and 118 were still alive) and 213 matched healthy controls (HCs) were enrolled. According to COUNT score and GNRI, patients with MSA had higher prevalence of malnutrition than HCs (44.6% vs. 14.1 and 17.9% vs. 0.9%, respectively). The median survival from symptom onset in patients with MSA in the malnutrition group was shorter than those in the normal-nutrition group (5.98 vs. 7.06 years, *p* = 0.012) by COUNT score. Additionally, malnutrition increased the risk of mortality in patients with MSA (HR = 1.556, *p* = 0.030) and MSA-P (HR = 1.973, *p* = 0.042) by COUNT score.

**Interpretation:**

Malnutrition was common in patients with early-stage MSA. Malnutrition increased the risk of mortality in patients with MSA, and early nutritional supplementation should be taken into consideration.

## Introduction

Multiple system atrophy (MSA) is an adult-onset, orphan, neurodegenerative disease characterized by a variable combination of autonomic failure, parkinsonism, cerebellar ataxia and pyramidal features (1). Meanwhile, MSA is a rapidly progressing disease with poor prognosis. The mean survival from the symptoms of onset is 6–10 years (2, 3), with few patients surviving more than 15 years (4). The symptomatic treatment is important, since no effective neuroprotective therapy is available at present.

Malnutrition was common in neurodegenerative disease, such as Parkinson’s disease (PD) and Alzheimer’s disease (5–8). Malnutrition was associated with worse disease status (6). Patients with MSA can develop malnutrition, which seriously affects the activities of daily living of patients with MSA (9). However, the prevalence of malnutrition in MSA remains largely unknown.

The Controlling Nutritional Status score (CONUT) score is a nutritional scoring tool that is calculated using serum albumin, total cholesterol level, and total lymphocyte count, representing protein reserve, energy consumption, and immune defense, respectively (10). The CONUT score is easily obtained from blood examinations, reflecting malnutrition because of the comprehensive assessment of nutritional status. The Geriatric Nutritional Risk Index (GNRI) is a simple tool, which requires routine measurement of albumin, weight, and height at admission (11). In recent years, CONUT and GNRI has been studied in PD. Nagano et al. enrolled 61patients with PD and measured their nutritional status using CONUT, they found that a poor nutritional status (i.e., CONUT score > 3) was significantly associated with a poor activity of daily living in patients with PD (12). Jiang et al. conducted a large-scale cross-sectional study with 1,478 PD patients and equal healthy controls (HC). The CONUT and GNRI were used for malnutrition stratification. The results shown that malnutrition was more prevalent in PD patients than that in HC (5). Recent studies indicated that malnutrition has prognostic potency concerning short- and long-term outcomes, including survival, in many diseases, particularly cancers and stroke (13–16). However, the predictive role of the malnutrition for mortality in patients with MSA has not been clearly established.

In the current study, we aimed to investigate the prevalence and prognostic role of malnutrition on mortality in patients with early-stage MSA using different malnutrition screen tools.

## Methods

### Study design and participants

**Figure 1:**
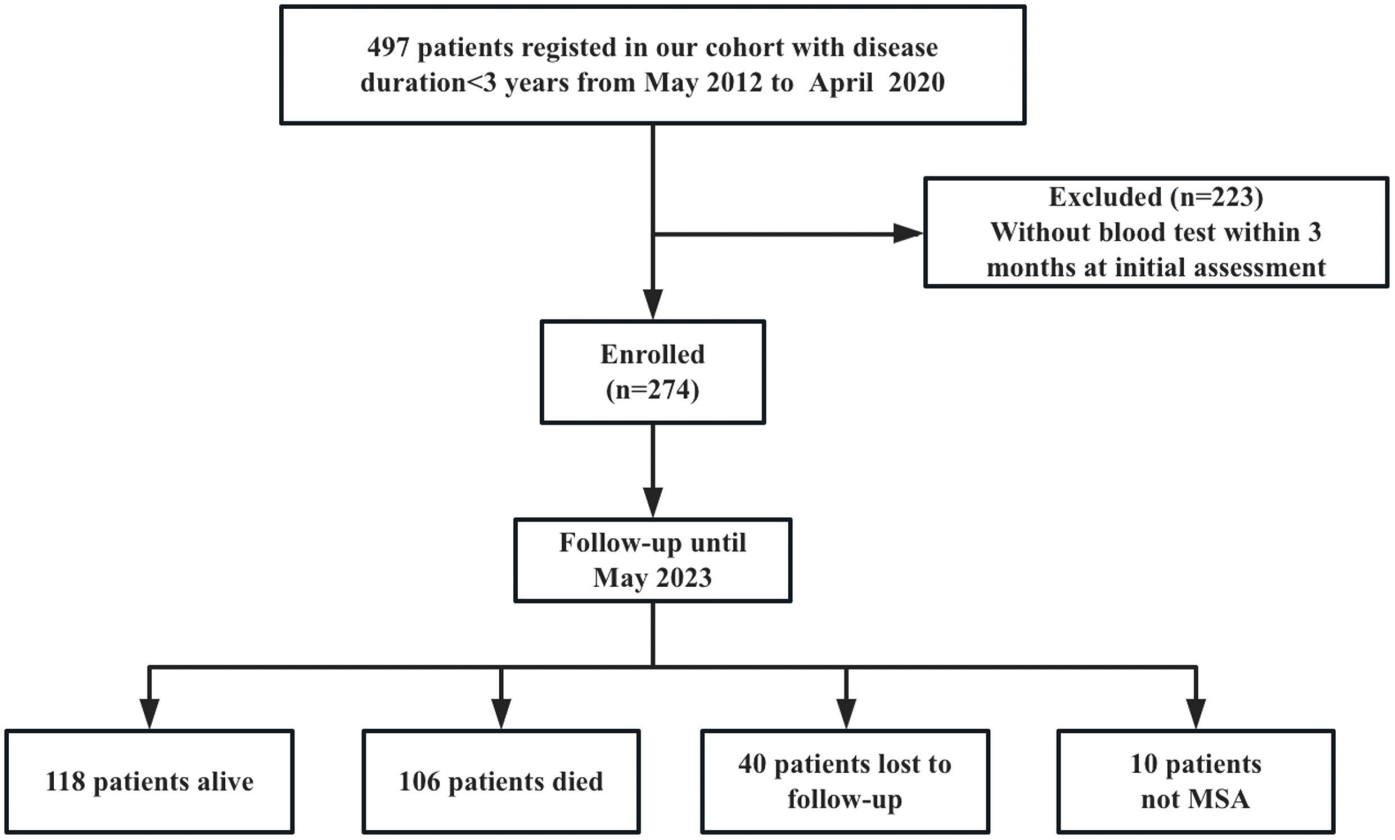
Study flow diagram (MSA, multiple system atrophy).

A total of 497 patients diagnosed with early phase MSA (disease duration<3 years) at the Department of Neurology, West China Hospital, Sichuan University, from May 2012 to April 2020, were recruited into the study. Patients with hepatic or renal failure, acute or chronic inflammatory diseases and infection at the initial assessment were excluded. All patients fulfilled the possible or probable MSA based on 2008 diagnostic criteria (17). Patients were screened for spinocerebellar ataxia (SCA) genes, including *SCA*1, −2, −3, −6, −7, to exclude the common forms of SCA. They also received brain MRI scans to exclude other neurological disorders. Patients were excluded if a blood test was not performed within 3 months of the initial clinical assessment (*n* = 223). Furthermore, 274 patients were followed every year by neurologists via telephone or in-person interviews. Until May 2023, forty patients were lost to follow-up, and 10 patients diagnosed with PD (*n* = 6), progressive supranuclear palsy (PSP) (*n* = 3), and dementia with lewy body (DLB) (*n* = 1) were excluded. Finally, 224 patients with a probable diagnosis of MSA were included in the final analysis (Figure 1). Additionally, 213 age- and sex-matched healthy controls (HCs) were enrolled. HCs were recruited from the medical examination center of West China Hospital of Sichuan University, who diagnosed with neuropsychiatric disorders, heart disease, hypertension, diabetes mellitus, hyperlipidemia, infectious diseases, renal dysfunction, or cancer were excluded.

### Demographic and clinical data collection

Patients with predominant parkinsonian features was defined as MSA-P, with predominant cerebellar ataxia was defined as MSA-C at the diagnosis. The clinical features of MSA-C include cerebellar ataxia, widespread gait, uncoordinated limb movements, action tremor and spontaneous or gaze-invoked nystagmus, while rigidity, bradykinesia, postural instability, a gait disorder tendency to fall and dysarthria predominate MSA-P (18). Additionally, these motor symptoms are often mixed, with cerebellar features in MSA-P and parkinsonian features in MSA-C. All patients were evaluated during in-person interviews with neurologists. Clinical information including sex, age, weight, height, age of onset, symptom of onset, disease duration, history of smoking and drinking, comorbidities, and treatments were collected. Disease duration was defined as the time from the date of symptom onset to the date of evaluation. Symptom of onset was defined as the initial presentation of any motor symptoms (i.e., parkinsonism or cerebellar ataxia) or autonomic features, with the exception of erectile dysfunction (17). Survival duration was defined as the interval from the date of symptom onset to the date of death for a deceased patient or from disease onset to the last follow up appointment for surviving patients. Death information was collected from the family reports.

### Evaluation protocol

Disease severity was rated on part I (activities of daily living, ADL), part II (motor examination), part III (autonomic examination), and part IV (global disability) of the Unified Multiple System Atrophy Rating Scale (UMSARS) (19). The total UMSARS score is the sum of parts I and II. Orthostatic hypotension (OH) was defined as a reduction in the systolic blood pressure by at least 30 mm Hg or diastolic blood pressure by at least 15 mm Hg after 3 min of standing up from a previous recumbent position of 3 min. BMI was calculated as body weight (kg) divided by height squared (m^2^).

### Hematological data collection

Blood sampling was performed after overnight fasting during the first assessment. Serum albumin value, total cholesterol and total lymphocyte count were tested in the Department of Laboratory Medicine, West China Hospital, Sichuan University.

CONUT score = serum albumin score + total cholesterol score + total lymphocyte count score (10). Serum albumin score (0, ≥3.5 g/dL; 2, 3.0–3.49 g/dL; 4, 2.50–2.99 g/dL; 6, <2.50 g/dL), total cholesterol score (0, ≥180 mg/dL; 1, 140–179 mg/dL; 2, 100–139 mg/dL; 3, < 100 mg/dL), and total lymphocyte count score (0, ≥1.6 × 10^9^ /L, 1, 1.20–1.59 × 10^9^ /L; 2, 0.80–1.19 × 10^9^ /L; 3, < 0.8 × 10^9^ /L). CONUT scores of 0–1, 2–4, 5–8, and 9–12 are considered to indicate normal nutritional status, mild malnutrition, moderate malnutrition, and severe malnutrition, respectively.

GNRI = 1.489*serum albumin (g/l) + 41.7*body weight(kg)/ideal body weight(kg). Ideal weight (kg) was calculated with the formula: 22*square of height (m2). If the actual body weight was more than the ideal body weight, the body weight- to-ideal body weight ratio was set to 1. A score > 98 was considered normal nutritional status, while 92–98, 82–91, and <82 indicate mild, moderate, and severe malnutrition, respectively.

### Ethics statement

Informed written consent was obtained from all participants. The study design was approved by the Ethics Committee of West China Hospital of Sichuan University.

### Statistical analysis

Patients with MSA were divided into the normal-nutrition group and the malnutrition group two groups according to CONUT score (<2 vs. ≥2) and GNRI (>98 vs. ≤98), respectively. All continuous data are presented as the mean ± standard deviation. All categorical variables are presented as numbers or percentages. For each continuous variable, we used the Kolmogorov–Smirnov test to evaluate the normality of the distribution. Student’s t-test or the Mann–Whitney U test was used to compare continuous variables between different groups. A chi-square test was performed to compare categorical variables. Kaplan–Meier survival analysis and log-rank test were used to analyze the prognostic significance of the CONUT score or GNRI for survival. Univariate and multivariate survival analyses were performed using the Cox proportional hazards regression model. In the multivariate Cox proportional hazards regression model, we adjusted for age (continuous variable), sex (male vs. female), subtype (MSA-P vs. MSA-C), symptom of onset (motor onset vs. autonomic onset), disease duration (continuous variable, in years), total UMSARS score (continuous variable), OH (yes vs. no), and urinary incontinence (yes vs. no). Statistical significance was set at *p* < 0.05. IBM SPSS software (version 22.0) was used for the statistical analysis.

## Results

### Baseline clinical characteristics

**Figure 2:**
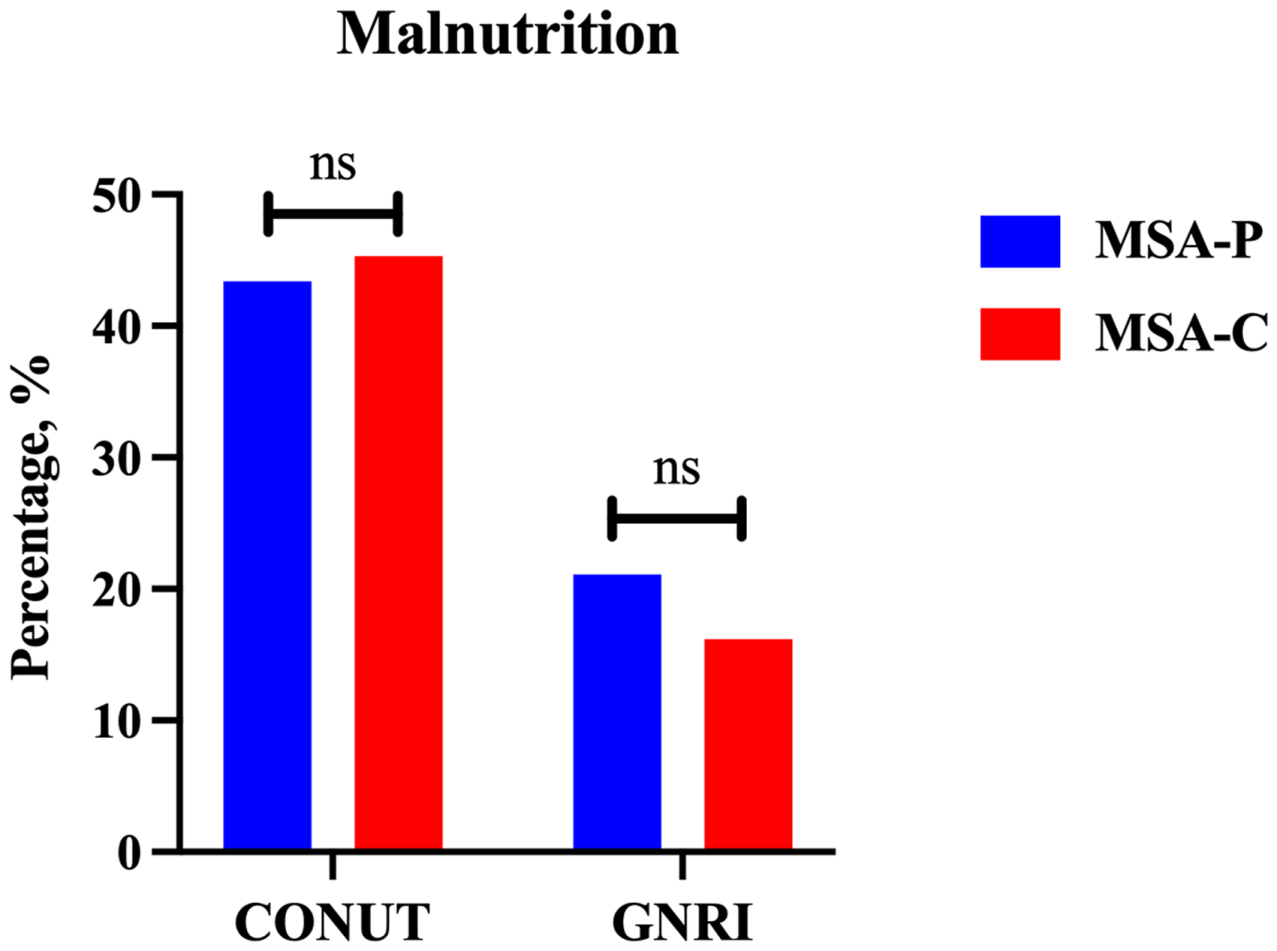
The comparison of prevalence of malnutrition between MSA-P and MSA-C (MSA-P, multiple system atrophy with predominate parkinsonism; MSA-C, multiple system atrophy with predominate cerebellar ataxia; CONUT, Controlling Nutritional Status; GNRI, Geriatric Nutritional Risk Index. ns: none of significance).

A total of 224 patients with MSA and 213 HCs were enrolled in the final analysis. The demographic and hematological data of the patients with MSA and HCs are shown in Table 1. Age, sex, and BMI were not significantly different between patients with MSA and HCs. Patients with MSA had lower levels of albumin (42.56 ± 3.48 vs. 46.49 ± 2.64 g/L, *p* < 0.001), total cholesterol (4.31 ± 0.89 vs. 5.29 ± 0.94 mmol/L, *p* < 0.001) and lymphocytes (1.71 ± 0.50 vs. 2.01 ± 0.65 10^9^/L, *p* < 0.001). According to COUNT score and GNRI, the prevalence of malnutrition in MSA was higher when compared to HCs (44.6% vs. 14.1 and 17.9% vs. 0.9%, respectively). There was no significant difference in the prevalence of malnutrition between patients with MSA-P and MSA-C based on COUNT score (43.4% vs. 45.3%, *p* = 0.792) and GNRI (21.1% vs. 16.2%, *p* = 0.371) (Figure 2).

**Table 1:** Demographic and the hematological data of MSA and HC.

Variables	Total MSA	HC	*p* value
Number	224	213	–
Age (*y*)	59.42 ± 7.95	59.56 ± 8.33	0.755
Sex (male, %)	105, 46.9%	102, 47.90%	0.832
BMI	23.36 ± 3.30	23.94 ± 3.05	0.059
Albumin(g/L)	42.56 ± 3.48	46.49 ± 2.64	<0.001*
Total cholesterol(mmol/L)	4.31 ± 0.89	5.29 ± 0.94	<0.001*
Lymphocytes(10^9^/L)	1.71 ± 0.50	2.01 ± 0.65	<0.001*
CONUT score	1.48 ± 1.23	0.63 ± 0.79	<0.001*
Malnutrition (%)	100, 44.6%	30, 14.1%	<0.001*
GNRI	103.55 ± 5.86	109.89 ± 4.65	<0.001*
Malnutrition (%)	40, 17.9%	2, 0.9%	<0.001*

MSA, multiple system atrophy; HC, healthy control; BMI, body mass index; CONUT, Controlling Nutritional Status; GNRI, Geriatric Nutritional Risk Index.

*Significant difference.

### Comparison demographic and clinical features between MSA with normal-nutrition and malnutrition

The demographic and clinical features of patients with MSA in the two groups according to CONUT score and GNRI are shown in Table 2. The mean age and age at onset of patients with MSA were 59.42 ± 7.95 and 57.83 ± 7.85 years, respectively. Among them, 33.9% of the patients were MSA-P and 46.9% of the patients were male. The mean disease duration was 1.83 ± 0.69 years at baseline.

**Table 2:** Comparison demographic and clinical features between MSA with normal-nutrition and malnutrition.

Variables		COUNT			GNRI		
	Total MSA	Normal-nutrition	Malnutrition	*p* value	Normal-nutrition	Malnutrition	*p* value
Number	224	124	100	–	184	40	–
Diagnosis subtype (MSA-P, %)	76, 33.9%	43, 34.7%	33,33.0%	0.792	60, 32.6%	16, 40.0%	0.371
Age (y)	59.42 ± 7.95	58.96 ± 7.42	60.00 ± 8.57	0.332	59.37 ± 7.90	59.66 ± 8.31	0.792
Age of onset (y)	57.83 ± 7.85	57.47 ± 7.51	58.28 ± 8.26	0.443	57.77 ± 7.76	58.08 ± 8.32	0.759
Sex (male, %)	105, 46.9%	51, 41.1%	54, 54.0%	0.055	90, 48.9%	15, 37.5%	0.190
BMI	23.37 ± 3.30	23.79 ± 3.25	22.84 ± 3.30	0.032*	23.92 ± 2.95	20.83 ± 3.66	<0.001*
Disease duration (y)	1.83 ± 0.69	1.87 ± 0.69	1.79 ± 0.68	0.381	1.81 ± 0.68	1.91 ± 0.73	0.312
Onset symptom (motor onset, %)	126, 56.3%	70, 56.5%	56, 56.0%	0.946	105, 57.1%	21, 52.5%	0.598
UMSARS-I	16.63 ± 6.49	16.55 ± 6.37	16.74 ± 6.67	0.856	16.23 ± 5.91	18.50 ± 8.52	0.211
UMSARS-II	18.55 ± 7.78	18.51 ± 7.36	18.60 ± 8.31	0.915	17.97 ± 7.26	21.23 ± 9.50	0.052
UMSARS-IV	2.12 ± 0.95	2.17 ± 0.93	2.06 ± 0.98	0.280	2.05 ± 0.90	2.45 ± 1.13	0.037*
Total UMSARS score	35.18 ± 13.58	35.06 ± 13.13	35.34 ± 14.19	0.877	34.20 ± 12.42	39.73 ± 17.48	0.120
OH (%)	71, 31.7%	37, 29.8%	34, 34.0%	0.506	59, 32.1%	12, 30.0%	0.799
Albumin(g/L)	42.51 ± 3.34	42.96 ± 3.13	41.96 ± 3.51	0.026*	43.44 ± 2.76	38.25 ± 2.32	<0.001*
Total cholesterol(mmol/L)	4.31 ± 0.89	4.80 ± 0.72	3.70 ± 0.68	<0.001*	4.40 ± 0.88	3.88 ± 0.84	0.001*
Lymphocytes(10^9^/L)	1.71 ± 0.50	1.93 ± 0.42	1.45 ± 0.47	<0.001*	1.73 ± 0.50	1.62 ± 0.52	0.154
Smoking (%)	69, 30.8%	37, 29.8%	32, 32.0%	0.728	63, 34.2%	6, 15.0%	0.017*
Drinking (%)	58, 25.9%	29, 23.4%	29, 29.0%	0.340	55, 29.9%	3, 7.5%	0.003*
Comorbidity							
Hypertension (%)	34, 15.2%	21, 16.9%	13, 13.0%	0.414	28, 15.2%	6, 15.0%	0.972
Diabetes mellitus (%)	23, 10.3%	12, 9.70%	11, 11.0%	0.746	20, 10.9%	3, 7.5%	0.727
Hyperlipidemia (%)	35, 15.6%	24, 19.4%	11, 11.0%	0.087	33, 17.9%	2, 5.0%	0.041*
Treatment							
Levodopa (%)	47, 21.0%	28, 22.6%	19, 19.0%	0.513	41, 22.3%	6, 15.0%	0.305
Amantadine (%)	14, 6.3%	7, 5.6%	7, 7.0%	0.677	11, 6.0%	3, 7.5%	1.000
Dopamine receptor agonist (%)	12, 5.4%	6, 4.8%	6, 6.0%	0.701	11, 6.0%	1, 2.5%	0.618
Buspirone hydrochloride (%)	14, 6.6%	9, 7.3%	5, 5.0%	0.488	12, 6.5%	2, 5.0%	1.000
Hypotensive drug (%)	28, 12.5%	18, 14.5%	10, 10.0%	0.310	22, 12.0%	6, 15.0%	0.598
Hypoglycemic agent (%)	19, 8.5%	11, 8.9%	8, 8.0%	0.816	16, 8.7%	3, 7.5%	1.000
Lipid-lowering drug (%)	15, 6.7%	7, 5.6%	8, 8.0%	0.483	14, 7.6%	1, 2.5%	0.411

MSA, multiple system atrophy; MSA-P, multiple system atrophy with predominate parkinsonism; BMI, body mass index; UMSARS, unified multiple system atrophy rating scale; OH, orthostatic hypotension; CONUT, Controlling Nutritional Status; GNRI, Geriatric Nutritional Risk Index.

*Significant difference.

According to the COUNT score, the normal-nutrition group had higher levels of BMI (23.79 ± 3.25), albumin (42.96 ± 3.13 g/L), total cholesterol (4.80 ± 0.72 mmol/L) and lymphocytes (1.93 ± 0.42 10^9^/L) compared to the malnutrition group (all *p* < 0.05). There were no significant differences in the age, diagnosis subtype, sex, disease duration, symptom of onset, OH, UMSARS-I, UMSARS-II, UMSARS-IV, total UMSARS score, history of smoking and drinking, comorbidities, or treatments between the two groups (all *p* > 0.05).

According to the GNRI, compared to the normal-nutrition group, the malnutrition group had lower levels of BMI (20.83 ± 3.66), albumin (38.25 ± 2.32 g/L), total cholesterol (3.88 ± 0.84 mmol/L), higher score of UMSARS-IV (2.45 ± 1.13), lower proportion of history of smoking (15.0%) and drinking (7.5%), lower proportion of hyperlipidemia (5.0%) (all *p* < 0.05).

### Survival analysis of CONUT score and GNRI in patients with MSA

**Figure 3:**
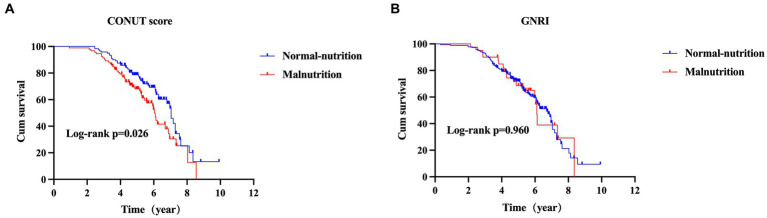
The comparison of survival between MSA with normal-nutrition and malnutrition by CONUT score **(A)** or GNRI **(B)** (MSA, multiple system atrophy; CONUT, Controlling Nutritional Status; GNRI, Geriatric Nutritional Risk Index).

At the end of the follow-up, one hundred and six (47.3%) patients with MSA died and 118 (52.7%) were still alive. As shown in Figure 3A, the survival duration was significantly different among the two groups (log-rank *p* = 0.012). The survival duration of patients in malnutrition group was shorter than those in normal-nutrition group (estimated median survival time: 5.98 (95% CI 5.33–6.64) vs. 7.06 (95% CI 6.72–7.36) years) according to the COUNT score.

According to the GNRI, the survival duration was not significantly different between patients in malnutrition group and normal-nutrition group (estimated median survival time: 6.11 (95% CI 5.97–6.26) vs. 6.69 (95% CI 6.09–7.29) years, log-rank *p* = 0.960) (Figure 3B).

Next, we performed the univariate and multivariable Cox proportional hazards regression analysis to demonstrate the predictive value of malnutrition on survival in MSA. After adjusting age, sex, subtype, symptom of onset, disease duration, total UMSARS score, OH, and urinary incontinence, the malnutrition was associated with an increased risk of mortality in patients with MSA by the CONUT score (HR = 1.556, 95%CI 1.043–2.322, *p* = 0.030) (Table 3). Additionally, the malnutrition increased the risk of mortality in patients with MSA-P after adjusting age, sex, symptom of onset, disease duration, total UMSARS score, OH and urinary incontinence by the CONUT score (HR = 1.973, 95%CI 1.025–3.799, *p* = 0.042) (Table 4). The malnutrition did not increase the risk of mortality in patients with MSA-C after adjusting age, sex, symptom of onset, disease duration, total UMSARS score, OH, and urinary incontinence by the CONUT score (HR = 1.408, 95% CI 0.826–2.399, *p* = 0.208) (Table 5). However, after adjusting the confounding factors, the malnutrition was not associated with poor survival in patients with MSA, MSA-P, or MSA-C by the GNRI (HR = 0.859, 95%CI 0.493–1.497, *p* = 0.592; HR = 1.568, 95%CI 0.704–3.491, *p* = 0.271; HR = 0.504, 95%CI 0.215–1.181, *p* = 0.115, respectively) (Tables 3–5).

**Table 3:** Univariate and multivariate Cox proportional-hazards regression analyses for survival in patients with MSA.

Variables	Univariate model		Multivariate model (CONUT)		Multivariate model (GNRI)	
	HR (95%CI)	*p* value	HR (95%CI)	*p* value	HR (95%CI)	*p* value
Age	1.014 (0.990–1.039)	0.256	0.995 (0.969–1.021)	0.706	1.003 (0.977–1.029)	0.824
Sex	1.210 (0.825–1.775)	0.330	1.239 (0.834–1.840)	0.288	1.289 (0.870–1.911)	0.206
Diagnosis subtype	1.317 (0.888–1.953)	0.171	1.028 (0.667–1.584)	0.902	1.010 (0.658–1.550)	0.964
Symptom of onset	0.963 (0.655–1.416)	0.848	0.960 (0.649–1.420)	0.838	0.947 (0.635–1.410)	0.787
Disease duration	0.908 (0.675–1.222)	0.524	0.600 (0.426–0.845)	0.003*	0.617 (0.439–0.867)	0.005*
Total UMSARS score	1.030 (1.016–1.044)	<0.001*	1.036 (1.020–1.053)	<0.001*	1.037 (1.021–1.054)	<0.001*
OH	1.314 (0.875–1.974)	0.187	1.291 (0.849–1.964)	0.232	1.323 (0.869–2.015)	0.192
Urinary incontinence	1.507 (1.025–2.216)	0.037*	1.419 (0.950–2.121)	0.087	1.379 (0.916–2.076)	0.124
Malnutrition (CONUT)	1.626 (1.108–2.385)	0.013*	1.556 (1.043–2.322)	0.030*	–	–
Malnutrition (GNRI)	1.013 (0.615–1.667)	0.960	–	–	0.859 (0.493–1.497)	0.592

MSA, multiple system atrophy; UMSARS, unified multiple system atrophy rating scale; OH, orthostatic hypotension; CONUT, Controlling Nutritional Status; GNRI, Geriatric Nutritional Risk Index.

*Significant difference.

**Table 4:** Univariate and multivariate Cox proportional-hazards regression analyses for survival in patients with MSA-P.

Variables	Univariate model		Multivariate model (CONUT)		Multivariate model (GNRI)	
	HR (95%CI)	*p* value	HR (95%CI)	*p* value	HR (95%CI)	*p* value
Age	0.989 (0.954–1.026)	0.559	0.978 (0.943–1.016)	0.250	0.979 (0.942–1.017)	0.265
Sex	0.694 (0.358–1.347)	0.280	0.816 (0.404–1.650)	0.571	0.851 (0.430–1.684)	0.643
Symptom of onset	1.442 (0.739–2.815)	0.284	1.608 (0.808–3.202)	0.176	1.564 (0.783–3.123)	0.205
Disease duration	0.960 (0.569–1.619)	0.878	0.727 (0.390–1.356)	0.316	0.744 (0.408–1.359)	0.337
Total UMSARS score	1.027 (1.007–1.048)	0.009*	1.028 (1.004–1.053)	0.024*	1.028 (1.002–1.054)	0.033*
OH	1.405 (0.729–2.707)	0.310	1.717 (0.863–3.416)	0.124	1.695 (0.838–3.431)	0.142
Urinary incontinence	0.963 (0.513–1.810)	0.908	0.924 (0.455–1.919)	0.832	0.951 (0.459–1.969)	0.893
Malnutrition (CONUT)	1.913 (1.025–3.571)	0.042*	1.973 (1.025–3.799)	0.042*	–	–
Malnutrition (GNRI)	2.096 (1.025–4.285)	0.042*	–	–	1.568 (0.704–3.491)	0.271

MSA-P, multiple system atrophy with predominate parkinsonism; UMSARS, unified multiple system atrophy rating scale; OH, orthostatic hypotension; CONUT, Controlling Nutritional Status; GNRI, Geriatric Nutritional Risk Index.

*Significant difference.

**Table 5:** Univariate and multivariate Cox proportional-hazards regression analyses for survival in patients with MSA-C.

Variables	Univariate model		Multivariate model (CONUT)		Multivariate model (GNRI)	
	HR (95%CI)	*p* value	HR (95%CI)	*p* value	HR (95%CI)	*p* value
Age	1.027 (0.996–1.060)	0.092	1.011 (0.974–1.049)	0.571	1.024 (0.988–1.062)	0.193
Sex	1.670 (1.023–2.728)	0.040*	1.501 (0.892–2.526)	0.126	1.443 (0.857–2.429)	0.167
Symptom of onset	0.771 (0.472–1.261)	0.300	0.789 (0.476–1.307)	0.357	0.743 (0.442–1.249)	0.262
Disease duration	0.851 (0.588–1.232)	0.393	0.528 (0.341–0.816)	0.004*	0.558 (0.361–0.863)	0.009*
Total UMSARS score	1.033 (1.012–1.054)	0.002*	1.039 (1.014–1.065)	0.002*	1.039 (1.014–1.064)	0.002*
OH	1.252 (0.742–2.112)	0.399	1.157 (0.654–2.046)	0.617	1.263 (0.717–2.226)	0.419
Urinary incontinence	1.817 (1.117–2.958)	0.016*	1.719 (1.038–2.846)	0.035*	1.524 (0.905–2.567)	0.113
Malnutrition (CONUT)	1.484 (0.911–2.419)	0.113	1.408 (0.826–2.399)	0.208	–	–
Malnutrition (GNRI)	0.577 (0.274–1.214)	0.147	–	–	0.504 (0.215–1.181)	0.115

MSA-C, multiple system atrophy with predominate cerebellar ataxia; UMSARS, unified multiple system atrophy rating scale; OH, orthostatic hypotension; CONUT, Controlling Nutritional Status; GNRI, Geriatric Nutritional Risk Index.

*Significant difference.

## Discussion

In this study, we found that malnutrition is common in patients with MSA and had a higher prevalence than HCs regardless of the COUNT score or GNRI. Additionally, the current study presents novel findings that CONUT score can be used as a predictive tool for mortality in patients with MSA, and malnutrition was significantly associated with poor survival in patients with MSA, particularly in the MSA-P subtype.

To our knowledge, this is the first study to explore the nutritional status of patients with MSA using the COUNT score and the GNRI. We found that patients with MSA had a higher prevalence of malnutrition than HCs regardless of the COUNT score or GNRI. Our previous study has reported that malnutrition was more prevalent in patients with PD than in HCs (5), which supported our results since MSA and PD belong to α-synucleinopathy. Additionally, it was determined that 44.6% of the patients with MSA were malnutrition according to the COUNT score, whereas 17.9% were malnutrition according to the GNRI. The prevalence of malnutrition assessed by GNRI (11.1%) was found to be lower than CONUT score (40.7%) in patients with PD (5). It has been reported that GNRI may be less reliable in detecting malnutrition in patients with PD (8). Thus, further studies will be needed to determine the usability of GNRI on patients with MSA. CONUT score is independent of age, disease duration, and disease severity of MSA in the early stage, which suggests that CONUT score is a good biomarker of nutrition in early-stage MSA. The causes of malnutrition are complex. It has been reported that age, BMI, drug use, motor and non-motor symptoms were associated with malnutrition in patients with PD (5). However, little is known about the causes of malnutrition in MSA. In our study, we found that MSA patients with malnutrition had a lower BMI and a severer disease severity, which was consistent with previous study (5). The drug use was not different between patients with and without malnutrition. This may be related to the lower dosage and fewer types of medication used by patients in the early stages of MSA. Thus, these drugs have may less impact on absorption of nutrients in early stage. However, further studies are needed to explore the factors related to malnutrition in early MSA. Patients with MSA suffer from malnutrition even at the early stage may be ignored, since it is believed that malnutrition is usually caused by dysphagia. Therefore, this study reminds us that malnutrition can occur at the early stage of MSA.

Additionally, poor nutritional status was associated with higher mortality in older adults (20). In a recent study, low albumin, protein, and hemoglobin, which indicate poor nutritional status, were associated with higher mortality in patients with MSA (21). And the low creatinine level, which reflect muscle wasting, significantly predicted higher mortality in MSA-P and not in MSA-C (21). The current study enrolled a relatively large cohort of early-stage MSA demonstrated that malnutrition at the baseline assessed by CONUT score was significantly associated with poor survival in patients with MSA, particularly in the MSA-P subtype. We failed to found that the malnutrition assessed by GNRI was associated with poor survival in patients with MSA, which may due to the relatively low prevalence of malnutrition detected by GNRI, and GNRI only contains the serum albumin and body weight. The malnutrition can also be detected in overweight or obese patients with PD (5). GNRI may underestimate the true prevalence of malnutrition in overweight/obese patients. Altogether, poor nutritional status in the early-stage of MSA was important in the prognosis of MSA.

The CONUT score is easily obtained and is calculated using objective values including serum albumin score, total cholesterol score, and total lymphocyte count score (10). Albumin is the most abundant plasmatic protein, and hypoalbuminemia is a mortality prognostic factor in elderly people (22). The level of serum albumin can be influenced by inflammatory state, particularly high concentrations of IL-6 and TNF-alpha (22). Additionally, serum albumin has specific antioxidant functions that due to its multiple ligand-binding capacities and free radical-trapping properties and are closely related to the structure and the redox state of the molecule (23). Additionally, low total cholesterol level was associated with poor survival in elder patients (24, 25). Cholesterol metabolism has been linked to immune functions (26). The immune system protects the body against harm by inducing inflammation, while uncontrolled inflammation is implicated in the pathogenesis of many human diseases, including allergy, chronic inflammation and cancer (27). Cholesterol metabolism has in both the induction and the resolution of the inflammatory response, and the pro- or anti-inflammatory duality of cholesterol metabolism is complex (27). Lymphocytes are involved in the regulation of immunity. The mechanism of adrenergic control of lymphocyte trafficking is involved in the diurnal variation of adaptive immune responses and the progression of inflammatory diseases (28). However, patients with MSA are in the state of inflammation activation at the early stage of the disease (29). Low levels of albumin, total cholesterol, and lymphocytes count may cause immune dysfunction, leading to poor prognosis of MSA. Those relevant mechanisms need to be further confirmed.

This study has several strengths. To our knowledge, the current study is the first to investigate the prognostic value of the CONUT score in patients with MSA. We highlight the predictive value of malnutrition at the early stage of MSA, which enables clinicians to identify MSA patients with malnutrition in the early-stage who may benefit from early nutritional supplementation.

This study has several limitations. First, some potential factors may affect immune nutritional status. However, we have reduced these potential factors as much as possible, since patients with hepatic or renal failure, acute or chronic inflammatory diseases and infection at the initial assessment were excluded. Second, this study was conducted in a single cohort at a single hospital. Third, all patients were clinically diagnosed without a postmortem diagnosis.

## Conclusion

In summary, we found that malnutrition is common in patients with MSA and had a higher prevalence than HCs regardless of the COUNT score or GNRI. Additionally, we demonstrated that malnutrition is associated with poor survival in patients with MSA, particularly in those with MSA-P. Early nutritional supplementation should be taken into consideration.

## Data availability statement

The datasets used and/or analyzed during the current study are available from the corresponding author on reasonable request.

## Ethics statement

The studies involving humans were approved by the Ethics Committee of West China Hospital of Sichuan University. The studies were conducted in accordance with the local legislation and institutional requirements. The participants provided their written informed consent to participate in this study.

## Author contributions

SL: conception and design of the study, statistical analysis, interpretation of data, and drafting manuscript. LZ: data collection and statistical analysis. YH, TY, CL, QW, RO, and XC: data collection. HS: study design, analysis and interpretation, and revision of the manuscript. All authors contributed to the article and approved the submitted version.
